# Clinical Lectures on the Diseases of Women and Children

**Published:** 1857-04

**Authors:** 


ART. VIII.— *Clinical Lectures on the Diseases of Women and Children.* By GUNNING S. BEDFORD, A. M., M. D., Professor of Obstetrics, Diseases of Women and Children, and Clinical Midwifery, in the University of New York. "Medicus curat morbos, natura sanat."—*Hippocrates.* Fourth edition, carefully revised and enlarged. New York: SAMUEL S. & WILLIAM WOOD. 1856.

Only a year since we bestowed our attention on the first edition of this pretty octavo. We recollect to have found much to censure and little to praise, and have seen nothing since in it to modify our opinions, unless it be that the profession at large, in greedily consuming four editions of it within a year, have failed to render a verdict in accordance with our notions.

In our obstinacy, however, we still insist that we are right; and that *if there has been* a large sale of this book, it is a fact which does no honor to the judgment of the profession. Four editions within a year constitute a powerful argument, but before we yield to it we shall ascertain how large were these several editions.

Certainly the republication of successive small issues is not to be classed among legitimate means of advertising.

